# Strain engineering and lattice vibration manipulation of atomically thin TaS_2_ films†

**DOI:** 10.1039/d0ra02499f

**Published:** 2020-04-28

**Authors:** Xing Wu, Yongqing Cai, Jihong Bian, Guohui Su, Chen Luo, Yaodong Yang, Gang Zhang

**Affiliations:** Shanghai Key Laboratory of Multidimensional Information Processing, School of Communication and Electronic Engineering 500 Dongchuan Road Shanghai 200241 China xwu@ee.ecnu.eud.cn; Institute of High Performance Computing (IHPC), A*STAR 138632 Singapore zhangg@ihpc.a-star.edu.sg; Frontier Institute of Science and Technology, State Key Laboratory for Mechanical Behavior of Materials, Xi'an Jiaotong University Xi'an 710054 China

## Abstract

Beside the extraordinary structural, mechanical and physical properties of two-dimensional (2D) materials, the capability to tune properties *via* strain engineering has shown great potential for nano-electromechanical systems. External strain, in a controlled manner, can manipulate the optical and electronic properties of the 2D materials. We observed the lattice vibration modulation in strained mono- and few-layer tantalum sulfide (TaS_2_). Two Raman modes, E_1g_ and E^1^_2g_, exhibit sensitive strain dependence, with the frequency of the former intensity increasing and the latter decreasing under a compressive strain. The opposite direction of the intensity shifts, which cannot be explained solely by van der Waals interlayer coupling, is attributed to strain-induced competition between the electron–phonon interlayer coupling and possible stacking-induced changes of the intralayer transport. Our results enrich the understanding of the lattice vibration of TaS_2_ and point to strain engineering as a powerful tool for tuning the electron–phonon coupling of 2D materials.

## Abstract 2

We observed lattice vibration modulation in strained mono- and few-layer tantalum sulfide. E_1g_ and E_2g_ exhibit sensitive strain dependence with the frequency of the former intensity increasing and the latter decreasing under a compressive strain.

## Graphical abstract

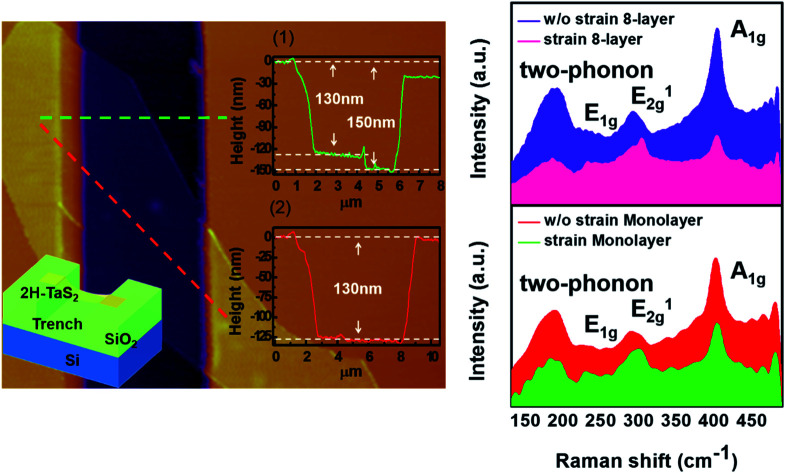

## Introduction

The two-dimensional (2D) transition metal dichalcogenide (TMD) materials including MX_2_ (M = Mo, W, Ta; X = S, Se, Te),^1–11^ are most important van der Waals materials that exhibit many interesting phenomena, such as charge density waves (CDWs), hidden phases and superconductivity.^12–15^ For the atomically thin tantalum sulfide (TaS_2_), the interaction between electron and phonon for a strong coupling enhanced superconductivity has attracted lots of research interest.^16,17^ The thickness dependent superconductivity feature has been reported in such types of layered materials, and the transition temperature *T*_c_ is found experimentally to be enhanced with decreasing the film thickness due to the interlayer interaction.^18–20^ The interlayer interactions in 2D materials are related to not only the thickness but also the layer stacking order. In this respect, the crystallographic structure of TaS_2_ crystals is generally different from the common structures of MoS_2_, MoSe_2_, WS_2_ and WSe_2_.^21,22^ According to the convention theory of Wilson and Yoffe,^21^ the position of the atoms in each TMD atomic plane can be specified by three points in a triangular lattice (A, B, C). The upper (lower) case denotes the chalcogenide (metal) atoms. 1 L TaS_2_ has the same trigonal prismatic (H) structure as 1 L MoS_2_ (denoted as ABA or equivalently ACA). As the layer number increases, however, the stacking order of TaS_2_ layers differs subtly from that of MoS_2_. Multilayer MoS_2_ exhibits the so called 2HC structure, which can be represented as (ABA or BAB). In contrast, TaS_2_ exhibits the 2HA structure (ACA BCB), in which the Ta atoms in all the layers are aligned vertically, but the sub-lattice is rotated by 60° with respect to that of the neighboring layer. It would be interesting to examine how this subtle stacking difference and the metallic nature of TaS_2_ may influence the interlayer interactions, especially under the compressive strain.

The nature of lattice vibration of material is fundamental in understanding its various physical properties, placing great demands on a comprehensive and complete determination of its elastic, electronic, phonon and electron–phonon properties. Moreover, it plays key role in various technological applications, including mobility in field-effect transistor,^23^ transition temperature of superconductor,^24^ performance of nano-electromechanical systems,^25^ and figure of merit of thermoelectric device.^26^ Raman spectroscopy is a powerful and non-destructive technique to investigate the lattice vibrational modes and interlayer interactions in 2D materials.^27^ In particular, recent research using ultralow-frequency Raman spectroscopy has revealed a set of interlayer phonon modes in few-layer graphene,^28–32^ phosphorene^13,15,33–35^ and MoS_2_, WS_2_, MoSe_2_ and WSe_2_.^27,36–40^ The shear (S) modes involves the lateral displacement of individual rigid layers. As these interlayer modes are created entirely from the interlayer coupling, they are highly sensitive to the detailed layer characteristics, including the layer number, interlayer coupling strength and stacking order, as well as surface and interface quality. Moreover, in application of 2D materials, strain can be introduced either intentionally or unintentionally. The difference in lattice constant and thermal expansion coefficient between 2D material and its supporting substrate can generate strain.^41–43^ And tensile strain also can be introduced in a controllable manner such as a tip of atomic force microscopes.^44^ Strain engineering, understood as the field that study how the physical properties of materials can be tuned by controlling the elastic strain fields applied to it,^45^ provides a perfect platform to manipulate the lattice vibration mode and electron–phonon coupling. More fascinating phenomena have been theoretically predicted for strained TaS_2_,^46^ but yet to be realized experimentally. Thus, strain controllable electron–phonon coupling measurements are critical for in-depth understanding and further applications of 2D TaS_2_.^47–49^ Comparing with other well studied TMDs such as MoS_2_, MoSe_2_, WS_2_ and WSe_2_, the phonon properties, interlayer interaction and strain dependences in monolayer and few-layer TaS_2_ are much less explored.

In this article, we present the first report of Raman scattering studies of mono- and few-layer 2H-TaS_2_ with and without strain. The compressive strain has been applied locally on the TaS_2_, while the lattice vibration has been detected by the dedicated designed ultrasensitive Raman spectroscopy and verified by the first-principle calculation.

## Experimental section

To prepare the suspended 2H-TaS_2_. High-quality 2H-TaS_2_ single crystal were grown Ta metal wires (99.95 purity) and S pellets (99.99% purity) by iodine (99.8%) vapour transport in a gradient of 730–770 °C in sealed quartz tubes for 21 days. The atomically thin suspended 2H-TaS_2_ membranes are obtained by using mechanical exfoliation method on the pre-defined trenches on 90 nm thick SiO_2_ wafers. The exfoliation process is carried out in the glove box with N_2_ atmosphere to avoid sample oxidation. The trenches are defined by standard photolithography method, followed by dry etching in an inductively coupled plasma (ICP) system, where CH_4_ and CHF_3_ are used as etching gases. The typical width of the trenches is 5 μm and the depth is ∼150 nm.

TaS_2_ flakes are imaged by atomic force microscopy (AFM) (Cypher S, Oxford Instruments Asylum Research, Inc., USA) to determine the sample thickness. The tapping mode of measurement (in the repulsive force regime) was chosen. For TaS_2_ samples exhibiting lateral thickness variation, we observed step heights of individual layers of 0.6–0.7 nm. This value is compatible with the 0.62 nm interlayer spacing of a single layer of the S–Ta–S building block of the TaS_2_ crystal. The measurements show that exfoliation produces layers with a discrete number of these units. We consequently designate the thickness of our films in terms of the number of these TaS_2_ layers (nL). From extensive AFM scanning of freshly deposited samples, we found no evidence of structural irregularity on the nanometer length scale.

Terahertz Raman spectroscopy was performed under normal incidence with a HeNe laser centered at 488 nm. The laser beam was focused to a diameter of ∼2 μm on the samples by a ×50 objective. The reflected radiation was collected by the same objective and analyzed with a grating spectrometer equipped with a liquid-nitrogen-cooled charge coupled device (CCD). A combination of one reflective Bragg grating and two Bragg notch filters removed the majority of the laser side bands and allowed measurements of the Raman shift down to ∼8 cm^−1^. The typical spectral resolution was 0.5 cm^−1^. To avoid significant laser heating of the samples, excitation powers of 1 mW (on the sample) were used. The heating effect was estimated to be <0.5 K under the excitation conditions for all samples.

First-principles calculations were performed to investigate the strain induced frequency shift of zone-centered phonons by using the Quantum-Espresso code.^54^ We used the norm-conserving pseudopotential and the local density approximation (LDA) of Perdew–Wang together with an energy cutoff up to 70 Ry. A 15 × 15 × 1 Monkhorst–Pack grid was adopted to sample the first Brillouin zone for the electronic densities. The relaxed in-plane lattice constants (*a* = *b*) for monolayer and bulk TaS_2_ are 3.254 and 3.252 Å respectively. The *c* lattice constant of bulk TaS_2_ is 11.598 Å. To avoid the spurious interaction between images, a vacuum region with thickness of 15 Å was used. The evolution of the frequencies of zone-centered phonons with strain was calculated within the scheme of density functional perturbation theory (DFPT).

## Results and discussion

It is challenging in experimental studying monolayer and few-layer TaS_2_, partially due to the difficulty in exfoliating and also particularly susceptible to oxidation in atmospheric conditions,^50^ which hinder the manipulation of atomic thin TaS_2_ flakes in air. Although complex encapsulation techniques help preserving samples from oxidation, we find that a rapid integration of freshly exfoliated flakes into final devices and their immediate transfer to vacuum conditions for measurement also permits retaining the pristine properties of most TaS_2_ samples (details see the method section). Benefitted from the appropriate transfer, clear Raman spectrum is observed in the present work.

**Fig. 1:**
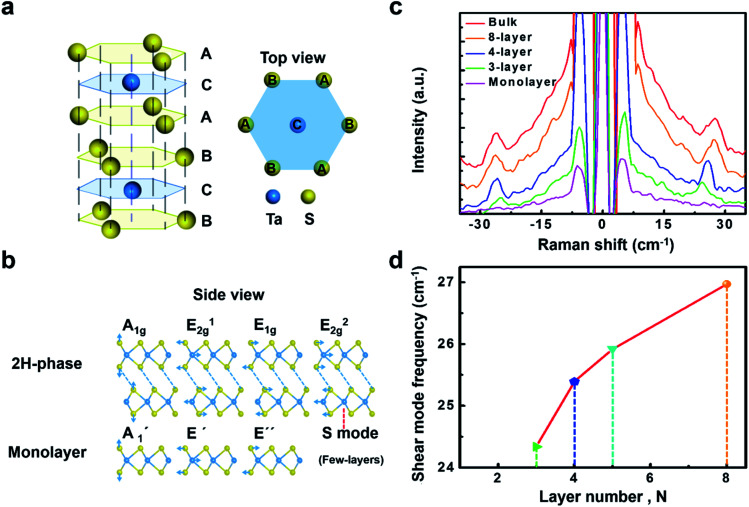
Characterization of the atomically thin TaS_2_ samples. (a) Trigonal prismatic structure of the monolayer TaS_2_ (left), and the corresponding honeycomb lattice formed by the Ta and S sub-lattices (right). (b) Schematic of the Raman active vibration mode of 2H-TaS_2_, including A_1g_, E^1^_2g_, E_1g_, and E^2^_2g_. (c) Raman spectra of bulk and atomically thin TaS_2_ at room temperature show the Stokes and anti-Stokes lines of the shear mode for layer thickness *N* ≥ 1. (d) Layer thickness *N* dependence of the shear mode frequency (symbols). The solid line corresponds to *ω*_S,Bulk_ cos(*π*/2*N*) with *ω*_S,Bulk_ = 26.7 cm^−1^.The spectra in (d) is displaced vertically for clarity.

Monolayer TaS_2_ consists of an atomic layer of Ta sandwiched between two layers of S in a trigonal prismatic structure (Fig. 1a). Bulk 2H-TaS_2_ is formed by stacking monolayer TaS_2_ with adjacent layers rotated by 180° with respect to one another. We summarize the characters of the phonon modes for both the bulk (2H phase) and monolayer TaS_2_ in Fig. 1b with respect to the symmetry assignment, frequency, optical character, and eigenvectors. The primitive cell of 2H-TaS_2_ and monolayer TaS_2_ contains six atoms. The Raman active modes are A_1g_, E^1^_2g_ and E_1g_. Out-of-plane A_1g_ Raman mode in 2H-TaS_2_, matches with the homopolar 
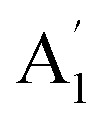
 mode in the monolayer counterpart, where the top and bottom sulfur layers vibrate out of phase with direction normal to the basal plane while the Ta layer remains stationary. In-plane vibration mode E^1^_2g_ involves displacement of Mo and S atom, associated with the in-plane vibration of two S atoms in opposite directions, and this mode is forbidden in backscattering measurements on a surface perpendicular to the *c* axis. Fortunately, this mode can be observed under the compressive strain although it disappears in strain-free samples (as discussed later). The low *ω*_S_ = 20–30 cm^−1^, which is strongly dependent on layer number N and is absent in monolayers (Fig. 1c), originates from interlayer shearing. With decreasing N, the shear mode frequency *ω*_S_ decreases quickly due to the reduced effective interlayer spring constant. It can be quantitatively described as *ω*_S_ = *ω*_S,Bulk_ cos(*π*/2*N*) with a bulk shear mode frequency *ω*_S,Bulk_ = 26.7 cm^−1^ (solid line, Fig. 1d), and has been used to accurately determine the layer number N of few-layer TaS_2_. Although TaS_2_ has different stacking order from MoS_2_, MoSe_2_, WS_2_ and WSe_2_, they share the same crystal symmetry groups and exhibit similar Raman selection rules for interlayer phonons. The sensitivity of the frequencies of these modes on the thickness suggests an additional method capable of determining the number of layer by Raman spectrum.

**Fig. 2:**
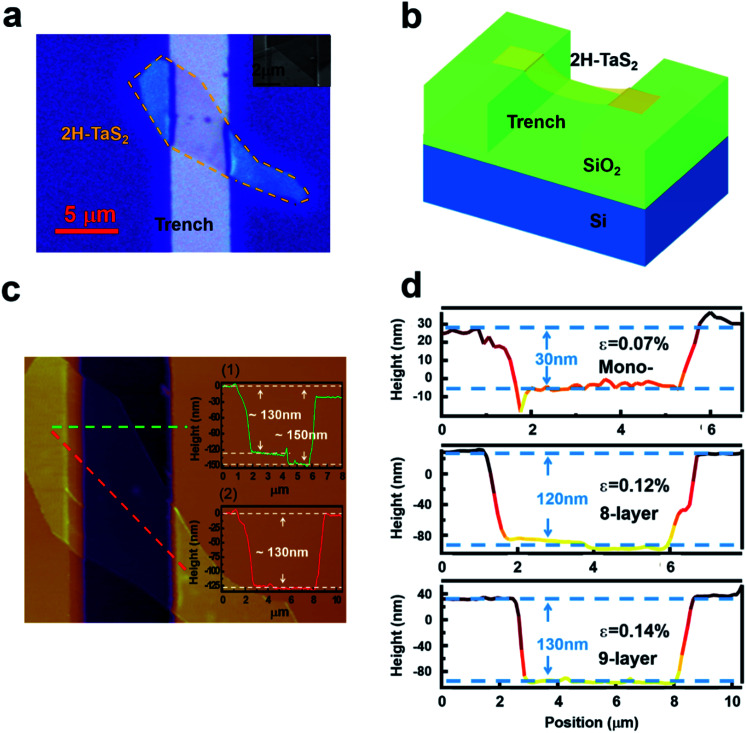
Suspended 2H-TaS_2_ flake. (a) Optical microscope image of an 8-layered suspended 2H-TaS_2_ flake device, which is transferred on a trench to introduce the local compressive strain. The scale bar is 5 μm. (b) Schematic of the setup of the suspended 2H-TaS_2_ flake. (c) Atomic force microscopy (AFM) image of the suspended 2H-TaS_2_ flake on the trench in (a). Insets: (1) line profile of the suspended 2H-TaS_2_ flake taken at the location of the green dotted line. The maximum deflection is 130 nm and the depth of the trench is ∼150 nm. It indicates that the flake is indeed suspended rather than touch the bottom of the trench. (2) Line profile of the suspended 2H-TaS_2_ flake taken at the location of the red dotted line. (d) The line traces of the topography (tip position) image of different 2H-TaS_2_ flakes from mono- to 9-layer with induced strain *ε* are 0.07%, 0.14%, and 0.16%, respectively.

To generate strain in a controllable manner, a tip of atomic force microscopes was adopted to load stress on suspended graphene.^44^ The other method is to transfer 2D material onto a flexible substrate, then strain can be generated by directly applying mechanical loading, for example, by bending, stretching or twisting the system.^51^ However, in both of these two strategies, only tensile strain can be loaded on 2D materials. To study the effect of compressive strain on lattice dynamics of atomically thin TaS_2_, a new strategy is developed in the present work. To study the strain effect of atomically thin TaS_2_, a special experimental setup is designed. Here in this work, by using advanced nano-fabrication process, local compression strain with a few micro meter has been applied to the 2H-TaS_2_ flake. The TaS_2_ is transferred on a trench to introduce the local compressive strain. The width of the trench is 5 μm. The advantage of this strategy is to use suspended atomically thin TaS_2_ sheets, suppressing the possible influence due to the substrate scattering. In our experiment, the compressive strain is fully applied on the TaS_2_ flake and is the intrinsic strain. Optical image shows dramatic contrast on the suspended area with the compressive strain and the one without the strain (Fig. 2a). It can be seen that the contrast of the suspended area is much lower than the one on the substrate. The schematic of the experiment platform is shown in Fig. 2b. It is noted that when a strain is applied to TaS_2_ materials, mechanical failure may occur either in the TaS_2_ materials or at the interface between TaS_2_ material and its supporting substrate. The Raman signals on the silicon dioxide substrate are consistent with the signals on other substrates, indicating no detachment failure at the interface between TaS_2_ and substrate. This is the same as the previous reports on the graphene system.^51^ Atomic force microscopy (AFM) has been used to verify the thickness and the depth of the TaS_2_ flake. Then, according to the geometry of our flakes, the compressive strain can be calculated by the equation:1
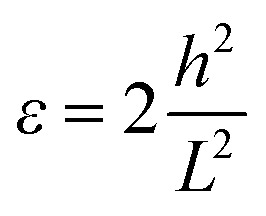
where *h* is the maximum deflection and *L* is the length of the suspended 2H-TaS_2_ flake (same as the width of the trench, around 5 μm). Here, *h* has been measured by AFM, as shown in the Fig. 2c. For the suspended 2H-TaS_2_ flakes, the applied compressive strains *ε* are 0.07%, 0.14%, and 0.16% (Fig. 2d).

**Fig. 3:**
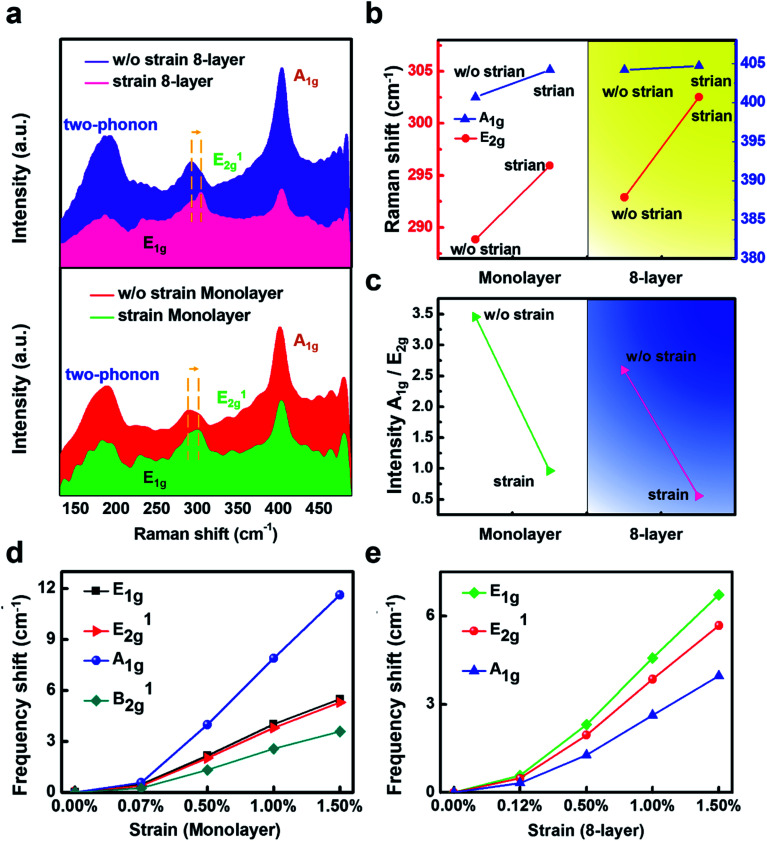
Strain dependence of Raman spectra of mono- and 8-layer 2H-TaS_2_ flakes. (a) Raman scattering intensity of mono- and 8-layer 2H-TaS_2_ with the compressive strain and without the strain, respectively. The blue arrow indicates the blueshift (stiffen) of E^1^_2g_ mode. The red dashed line shows the E_1g_ mode appears clearly from the strained flake. (b) The frequency shifts of E^1^_2g_ and A_1g_ modes for mono- and 8-layer 2H-TaS_2_ flake with the compressive strain and without the strain. (c) Raman intensity ratio between the A_1g_ peak and E^1^_2g_ peak extracted from (a). (d) & (e) Frequency shift for optical modes under the compressive strain in the flakes calculated by the first-principles calculation within the framework of density functional perturbation theory (DFPT). The opposite direction of the intensity, which cannot be explained solely by van der Waals interlayer coupling, is attributed to strong electron–phonon interactions.

Representative Raman spectra for mono- and 8-layer TaS_2_ at room temperature are shown in Fig. 3a. Among the four Raman-active modes of the bulk TaS_2_ crystal, the prominent features observed below 500 cm^−1^ include two-phonon peak at ∼185, E^1^_2g_ ∼ 290, and A_1g_ ∼ 404 cm^−1^. It is found that mono- and few-layer TaS_2_ flakes under compressive strain exhibit a strong in-plane vibrational mode at ∼230 cm^−1^, corresponding to the E_1g_ mode. In contrast, this mode was not observed in earlier studies of mono and few-layer TaS_2_ without strain.^18^ In strain-free TaS_2_, E_1g_ mode is absent attributed to the existence of trigonal prismatic coordination, which renders the vibration Raman inactive. This Raman behavior of TaS_2_ still resemble of MoS_2_. Although the detailed atomic configurations of TaS_2_ and MoS_2_ are different, the two 2H polytypes share the same symmetry point groups — *D*_3d_ group with inversion symmetry for even layer number, and *D*_3h_ group with mirror symmetry for odd layer number.^52^ Obvious stiffen (blueshift) of E^1^_2g_ signal could be observed, from 289 cm^−1^ to 296 cm^−1^ in monolayer and from 293 cm^−1^ to 303 cm^−1^ in 8-layer samples, while the other peaks remain unchanged with the compressive strain. The peak width is broaden as well, and this is different from the previous prediction that the compression would introduce enhancement of the intensity of the E^1^_2g_ mode.

We further confirm the mode assignment by calculating the phonon frequencies in mono- and bulk TaS_2_ using density functional perturbation theory (DFPT). It is interesting to find that all the modes show a positive value of *γ*, indicative of a normal behavior of stiffen frequencies with shrinking the lattice host. The variation of the frequencies for modes at the *Γ* point with compressive and tensile strains is plotted in Fig. 3(d) and (e), where the frequency shift (*δ*) with strain (*ε*) is defined as *δ* = *ω*(*ε*) − *ω*(0). The different slopes of the *δ*–*ε* curves reflect the different stiffening or softening behavior of each phonon mode under strain. For the 
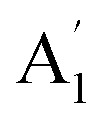
 mode, the slope is the largest among all the modes for the monolayer, while the smallest for the bulk. For the bulk case, the calculation results are consistent with the experiments. But the monolayer result is different from the experiment results. This paradox could be due to the strong electron–phonon coupling in the monolayer TaS_2_, while the calculation only considers the vdW coupling. Generally speaking, the eigenvector of this mode shows that the S atoms vibrate in counterphase in direction normal to the plane (Fig. 1) and the Ta plane remains stationary. Thus the frequency is sensitive to the in-plane strain, but relatively insensitive to the disturbance normal to the plane such as the compressive strain, electronic doping, or chemical doping above the planes. In contrast, the E′ mode involves the in-plane vibration, and thus is more sensitive to the in-plane strain. Note here that the trends for these two modes are reversed in the case of doping on the layer, where the 
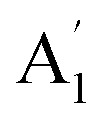
 mode shows significant softening behavior, whereas the E′′ mode remains nearly constant. In comparison to 2H-MoS_2_, our study of the larger slope of the 
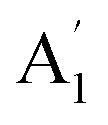
 mode (E^1^_2g_ for bulk) than the E′ mode (A_1g_ for bulk) with strain is different from previous measurements. This is probably due to the ABA and ABC stacked 2D materials show that the Raman activity of the interlayer modes is highly sensitive to the stacking-induced changes of the intralayer bonding. The opposite direction of the intensity, which cannot be explained solely by van der Waals interlayer coupling, is attributed to strong electron–phonon interactions.

For all the flake thickness, with and without the compressive strain, the out-of-plane A_1g_ vibration remained unchanged. This is unusual compared with other 2D TMDs such as MoS_2_, which A_1g_ increases with the thickness. Within a classical model for coupled harmonic oscillators, the E^1^_2g_ and A_1g_ modes are expected to stiffen as additional layers are added to form the bulk material from individual layers, since the interlayer vdW interactions increase the effective restoring forces acting on the atoms. While the unchanged A_1g_ mode observed in our measurements with the compressive strain disagrees with this prediction. The failure of the model could reflect the presence of additional interlayer interactions; it could also indicate that the implicit assumption that stacking order affects intralayer bonding is incorrect. In addition, as the compressive strain increases, the A_1g_ mode does not change shows that even the nominally interlayer interaction in TaS_2_ cannot affect intralayer bonding and lattice dynamics. To further study the changes of the Raman peaks-dependent strain effect, we extract the data from the Fig. 3a to show the detailed results (the frequency shift of E^1^_2g_ and A_1g_ mode for mono- and 8-layer 2H-TaS_2_ flake with and without the strain) in Fig. 3b. In addition, Raman intensity ratio between the A_1g_ peak and E^1^_2g_ peak is plotted in Fig. 3c to demonstrate the repeatability of the strain effect.

**Fig. 4:**
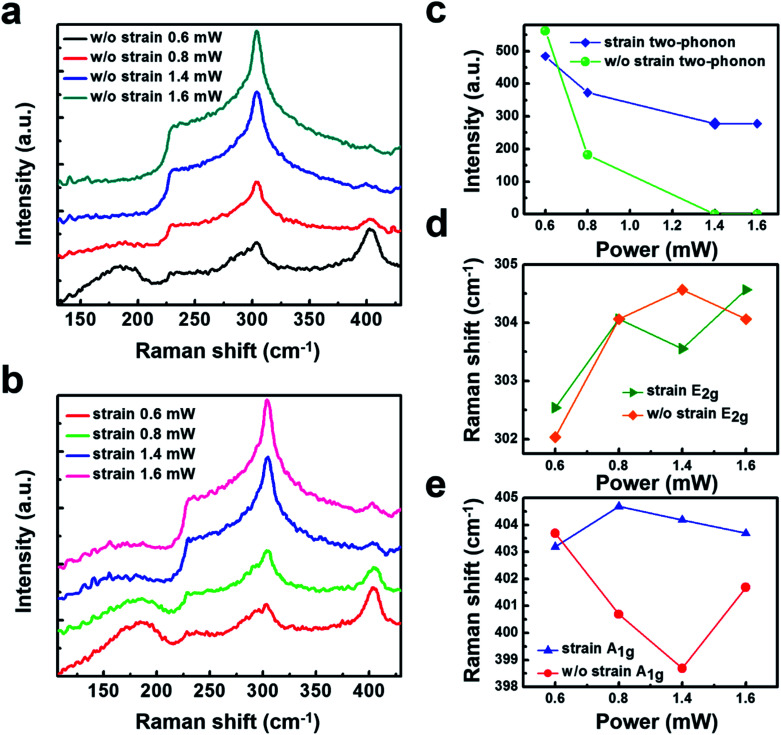
Raman characteristic of the suspended 2H-TaS_2_ flake under different power of the laser beam: (a) without the compressive strain, and (b) with the compressive strain. Raman spectra of the two-phonon peak fade away with a high irradiation power of the laser beam. But the suspended 2H-TaS_2_ flake impedes the phenomenon in (a). (c) Raman intensity of the two-phonon peak extracted from (a) and (b). (d) Raman shift of the (d) E^1^_2g_ peak, and (e) as a function of different powers of the laser beam.

It is important to highlight that as for some TMDs are sensitivity to the Raman laser beam. To understand the influence of the irradiation power impacts on the sample, low irradiation power of 0.6 mW, 0.8 mW, and the high irradiation power of 1.4 mW, 1.6 mW are selected. The Raman spectra are tested on a normal and suspended 2H-TaS_2_ flake. Under the high irradiation power, the Raman spectra of the two-phonon peak fades away, as shown in Fig. 4a. It is interesting to find that the two-phonon peak in Fig. 4a disappears in the suspended 2H-TaS_2_ while still exists in Fig. 4b when the compressive strain is on. Our measurement gives further physical insights that the strain can impede the phenomenon that the two-phonon peak fade away under a high irradiation power. To examine more carefully of the two-phonon peak shape, Raman intensity of the two-phonon peak under the selected irradiation power is shown in Fig. 4c. It can be clearly observed that when the irradiation power is up to 1.4 mW, the sample with strain has a strong decrease that the two-phonon intensity becomes to almost zero. By contrast, the appearance of a strong relative intensity suggested that the suspended 2H-TaS_2_ flake still has a two-phonon peak. Fig. 4c shows that the blue-shift of E^1^_2g_ under the strain. Analysis the A_1g_ mode with different irradiation powers has also been done, and it is found that a slight fluctuation in the two samples. These results have been tested repeatedly as shown in ESI Fig. S1–S3.† As the increase of the laser beam power, the intensity of E^1^_2g_ mode increases, while the one in A_1g_ mode deceases. Similar suppression of the A_1g_ mode has been observed in few-layer graphene due to the damping caused by surface adsorbates under high temperature.^53^

## Conclusions

In summary, we have reported the important Raman signature variety of atomically thin 2H-TaS_2_ layers under various conditions, such as different stress-deformation and irradiation power. In contrast to the regular 2H-TaS_2_ sample, we find that the Raman signature for 2H-TaS_2_ sample with a stress-deformation can generate blue-shift of E^1^_2g_ mode peak, enhance the E_1g_ mode and impede the two-phonon peak fade away under a higher irradiation power. Our results enrich the understanding of electronic structure of atomically thin 2H-TaS_2_, demonstrated the possibility to adjust the vibration modes of atoms *via* strain engineering, which extend their potential applications on nano-electromechanical systems (NEMS) and flexible electronics.

## Author contributions

X. W. and G. Z. conceived and designed the experiments, fabricated the suspended samples and carried out the Raman experiments. G. Z. and Y. Q. C. carried out numerical simulations and interpretation. G. H. S., Y. D. Y. and J. H. B. carried out AFM measurements. All authors contributed to the discussion and commented on the manuscript.

## Conflicts of interest

There are no conflicts to declare.

## Supplementary Material

RA-010-D0RA02499F-s001
