# Supplementary material for: Strain engineering and lattice vibration manipulation of atomically thin TaS2 films
Source: RSC Adv. 2020 Apr 28;10(28):16718–26. doi: 10.1039/d0ra02499f (PMC9053043; doi:10.1039/d0ra02499f)
Supplement: RA-010-D0RA02499F-s001 [file RA-010-D0RA02499F-s001.pdf]

Electronic Supplementary Material (ESI) for RSC Advances.
This journal is © The Royal Society of Chemistry 2020

Supplementary Materials

Strain engineering and lattice vibration manipulation of atomically thin TaS_2 films

Xing Wu[1,#,*], *Yongqing Cai*[2,#], *Jihong Bian*[3,#], *Guohui Su*[1], *Chen Luo*[1], *Yaodong Yang*[3], *Gang Zhang*[2,*]

[1] Shanghai Key Laboratory of Multidimensional Information Processing, School of Communication and Electronic Engineering, East China Normal University, 500 Dongchuan Road, Shanghai 200241, China.

[2] Institute of High Performance Computing (IHPC), A*STAR, Singapore 138632, Singapore.

[3] Frontier Institute of Science and Technology, State Key Laboratory for Mechanical Behavior of Materials, Xi'an Jiaotong University, Xi'an 710054, China.

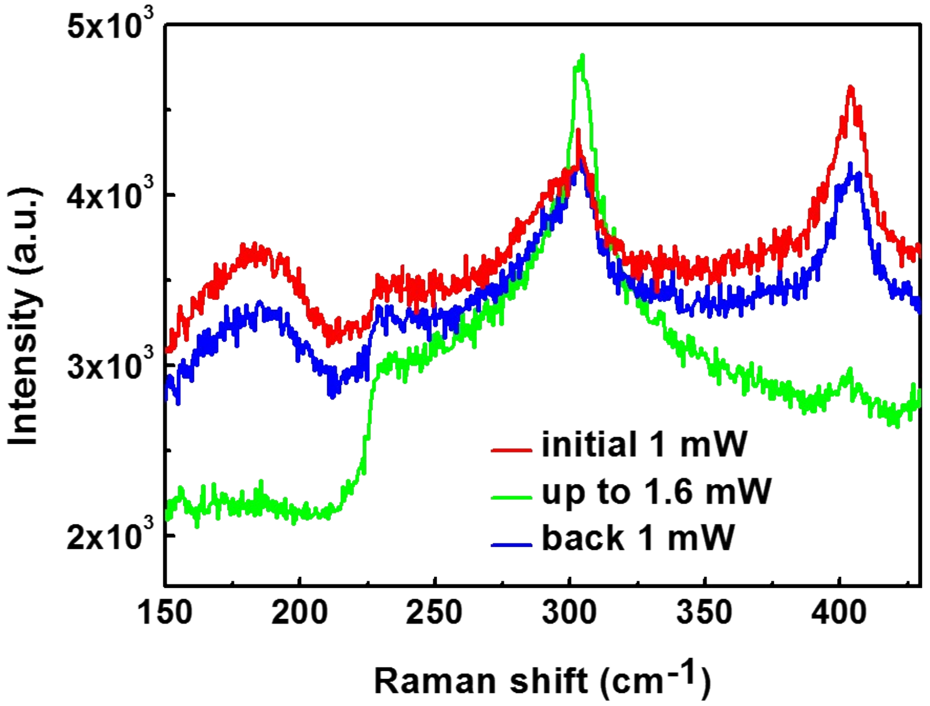

Figure S1: Raman spectra of the suspended 2H-TaS_2 flakes under different laser beam power values from 1 mW, 1.6 mW, and back to 1 mW. After irradiated under high laser beam power, the TaS_2 flake remains untainted, indicating the laser power of 1 mW used in the experiment introduces no additional destruction of the samples.

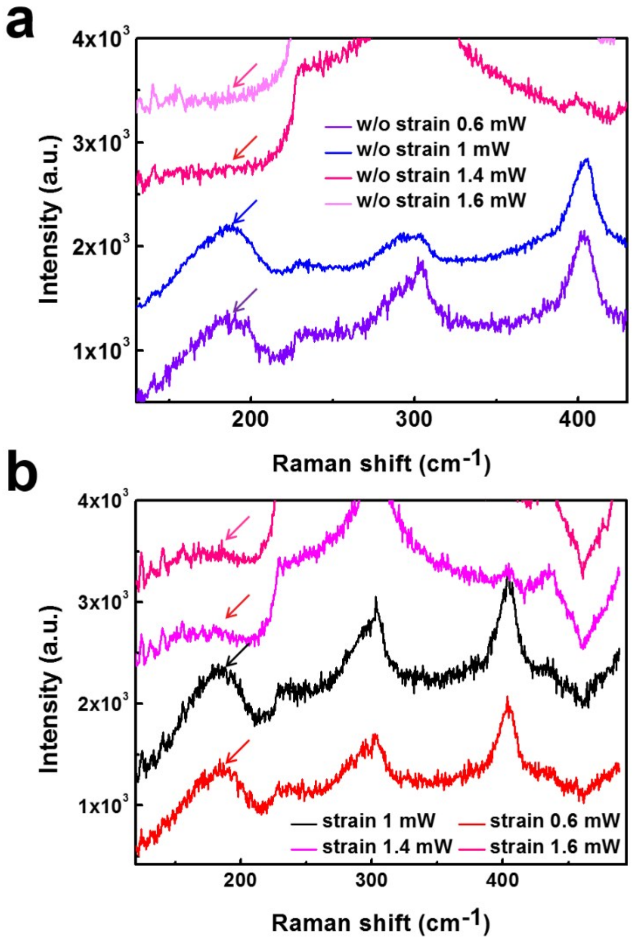

Figure S2: Raman spectra of 2H-TaS_2 under different laser beam power values with and without the compressive strain. (a) Irradiation power ranging from 0.8 mW to 1.6 mW, the two-phonon peak is suppressed above 1 mW, as indicated by the arrows. (b) Raman spectra collected on the strained 2H-TaS_2 flake. The two-phonon peak remains even above 1 mW, which is different from the unstrained samples. The two-phonon mode is enhanced with the compressive strain.

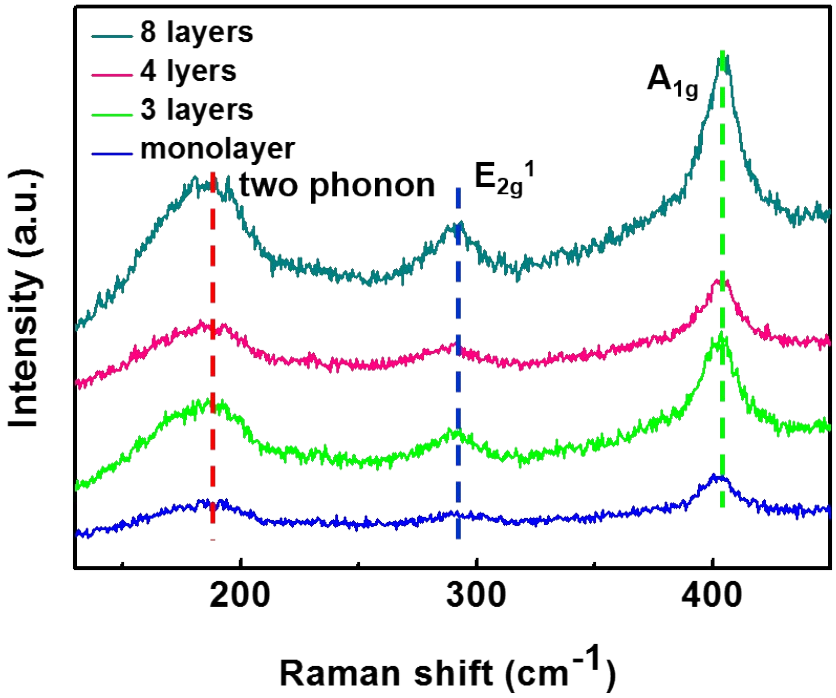

Figure S3: Thickness dependence of the unstrained 2H-TaS_2 flake. There is no obvious change in the E_{2g}^1 and A_{1g} as the thickness increases. In comparison to MoS_2, these two modes are highly thickness depended.

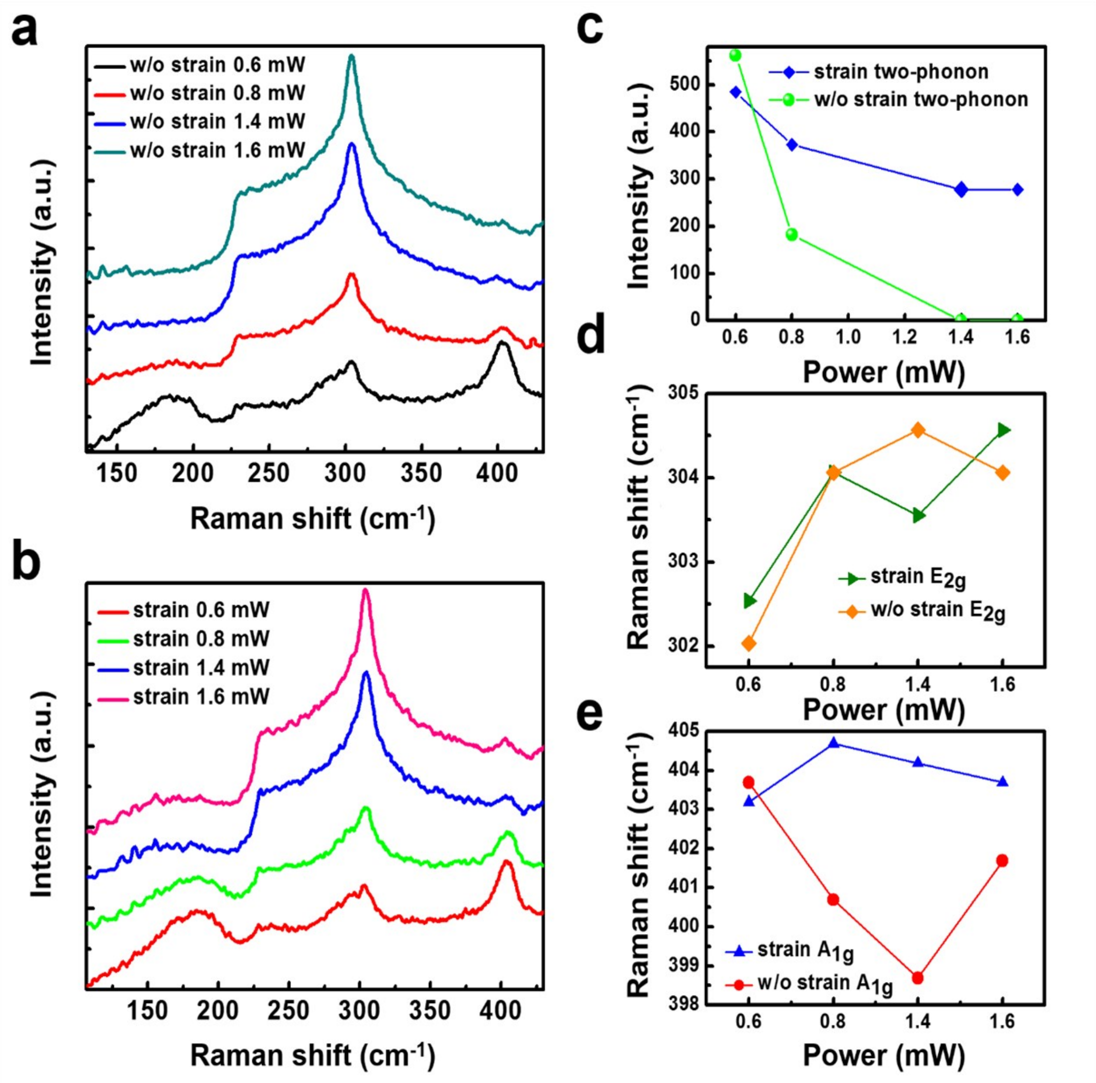

Figure 4. Raman characteristic of the suspended 2H-TaS_2 flake under different power of the laser beam:(a) without the compressive strain, and (b) with the compressive strain. Raman spectra of the two-phonon peak fade away with a high irradiation power of the laser beam. But the suspended 2H-TaS_2 flake impedes the phenomenon in (a). (c) Raman intensity of the two-phonon peak extracted from (a) and (b). (d) Raman shift of the (d) E_{2g}^1 peak, and (e) as a function of different powers of the laser beam.
